# Treatment of unstable C1 semi-ring fractures with direct C1 pedicle screw fixation using a navigational template: A case report and literature review

**DOI:** 10.1097/MD.0000000000033800

**Published:** 2023-05-17

**Authors:** Wei-Xin Dong, Zhen-Tao Chu, Yong Hu

**Affiliations:** a Department of Spinal Surgery, Ningbo No.6 Hospital, Ningbo, Zhejiang Province, China.

**Keywords:** fractures, navigational template, pedicle screw

## Abstract

Because of the risk of C1 to C2 instability, which would reduce the mobility of the occipito-atlanto-axis articulation, unstable C1 semi-ring fractures are typically treated with C1 to C2 or C0 to C2 fusion. The vertebral artery and spinal cord are at risk of harm during the installation of C1 pedicle screws. There is a need for a method that can maintain the occipito-atlanto-axis articulation’s mobility and increase the safety of C1 pedicle screw fixation, particularly for surgeons who have less experience inserting C1 pedicle screws freehand.

**Patient concerns:**

A 45-year-old man who had suffered a severe fall from a height of 2.5 m presented with pain in his cervical spine. Magnetic resonance imaging and computed tomography were used to diagnose unstable atlas fractures.

**Diagnosis:**

According to radiographic studies, the patient had a unilateral anterior and posterior arch fracture (semi-ring fracture, Landells type II), as well as fractures and transverse ligament avulsion at the attachment site.

**Interventions:**

We fixed the C1 directly with a pedicle screw using a navigational template.

**Outcomes:**

Both during and after the operation, there were no connected complications. Imaging at 12 months after surgery demonstrated that the fracture had united. The average visual analog scale score decreased from 8 before surgery to 2.

**Lessons:**

In particular for surgeons with less experience placing freehand C1 pedicle screws, direct C1 pedicle screw fixation with the aid of a navigational template was a good option because it can preserve the mobility of the occipito-atlanto-axis articulation and improve the safety of C1 pedicle screw.

## 1. Introduction

Fractures of the atlas have been reported to account for 25% of craniocervical injuries, 3 to 13% of cervical spine injuries, and 1.3 to 2% of all spinal injuries.^[1,2]^ The unique anatomy of the C1 ring presents many challenges for the treatment of C1 fractures compared to other cervical spines. Although the fact that the majority of atlas fractures can be treated conservatively with a hard collar or halo-vest, the clinical outcomes are sometimes unpredictable, and patients frequently experience malunion or nonunion.^[3]^ When an atlas fracture is unstable, C1 to 2 fusion or C0 to 2 fusion is typically used for safety. Atlantoaxial joint function will be lost as a result of this treatment, and the chance of adjacent segment degeneration will also rise.^[4,5]^ Given these flaws, a better-optimized treatment will considerably improve the clinical result. The following case report details an alternative surgical technique that uses direct pedicle screw fixation for C1 semi-ring fractures, assisted by a navigation template. This surgical method aims to preserve the postoperative mobility of the atlantoaxial spine, reduce the difficulty of pedicle screw implantation, and improve the accuracy and safety of screw insertion.

## 2. Case report

**Figure 1.**
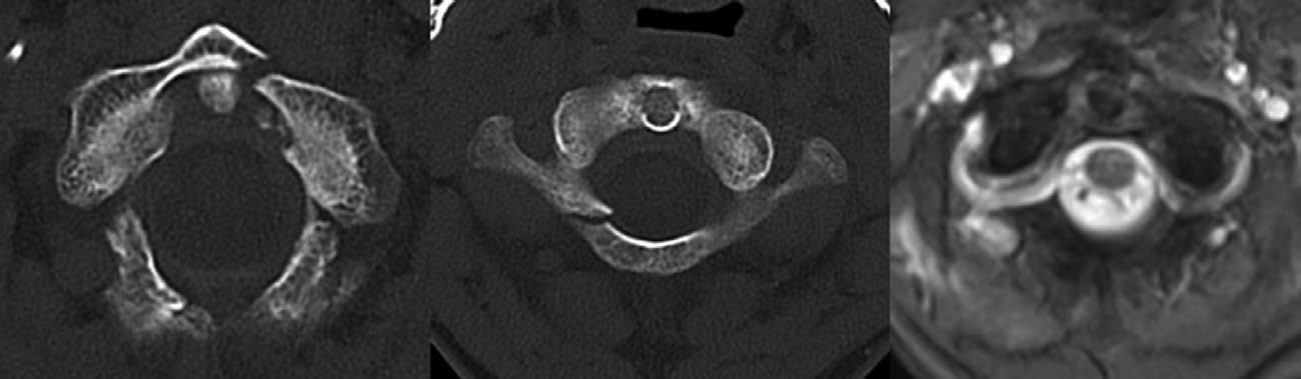
CT scan revealed unilateral anterior and posterior arch fracture (Landells type II semi-ring fracture). MR scan showed fractures and avulsion at the transverse ligament was attached. CT = computed tomography, MR = magnetic resonance.

A 45-year-old man, with no prior history of the lesion, presented to our emergency department complaining of pain in his cervical spine for 2 hours following a 2.5 m fall. A physical examination found tenderness in the cervical spine but no neurological deficits. The VAS score prior to surgery was 8. Initial computed tomography (CT) scan revealed unilateral anterior and posterior arch fracture (Landells type II semi-ring fracture) (Fig. 1). A magnetic resonance scan later confirmed fractures and avulsion at the location where the transverse ligament was attached (Fig. 1). The patient’s cervical spine was stabilized with a cervical brace after admission to prevent further movement. Treatment options discussed with him include halo immobilization, C1 to 2 fusion, direct fixation with pedicle screws, or direct pedicle screw implantation assisted by a navigation template. The patient chose to treat C1 semi-ring fractures with pedicle screws assisted by a navigation template in consideration of their expectations for functional recovery and surgical safety. And inform the patient of any possibility that C1 to 2 bone graft fusion may be required due to atlantoaxial instability in the long term.

**Figure 2.**
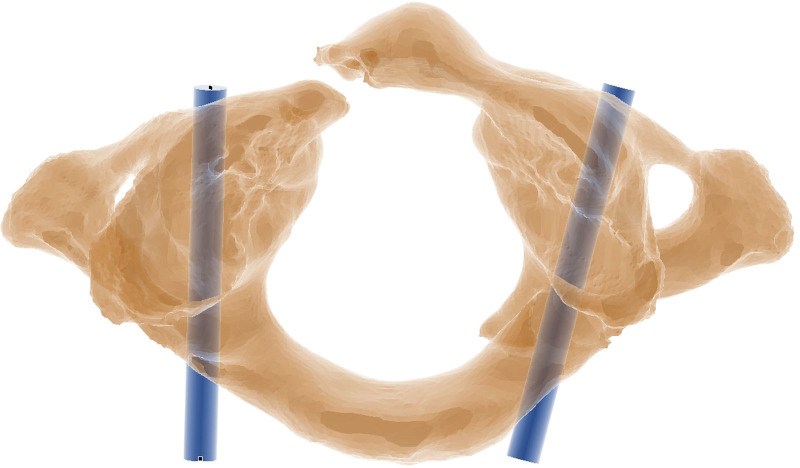
The ideal trajectory of C1 pedicle screws for a patient with an atlas fracture.

In this case, 5 kg of inline traction was used to achieve initial stability and alignment. Mimics software 21.0 (Materialise, Leuven, Belgium) was used to reconstruct the 3-dimensional atlas models, and the optimal trajectory for C1 pedicle screws was planned to reduce risk based on the corresponding anatomical structure of the patient (Fig. 2). After that, a rapid prototyping device materialized the drill guide template and used low-temperature plasma to sterilize it.

**Figure 3.**
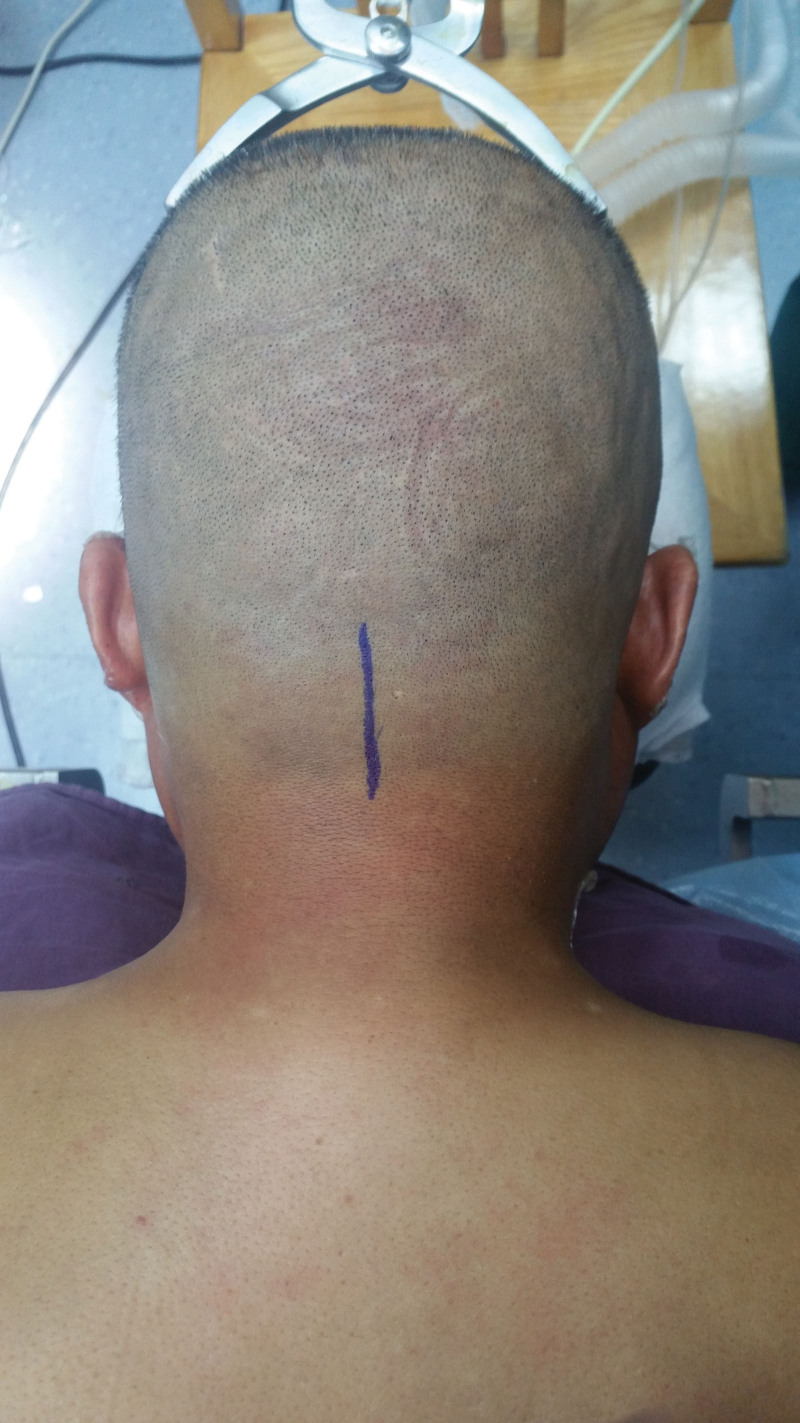
Planned surgical incision.

**Figure 4.**
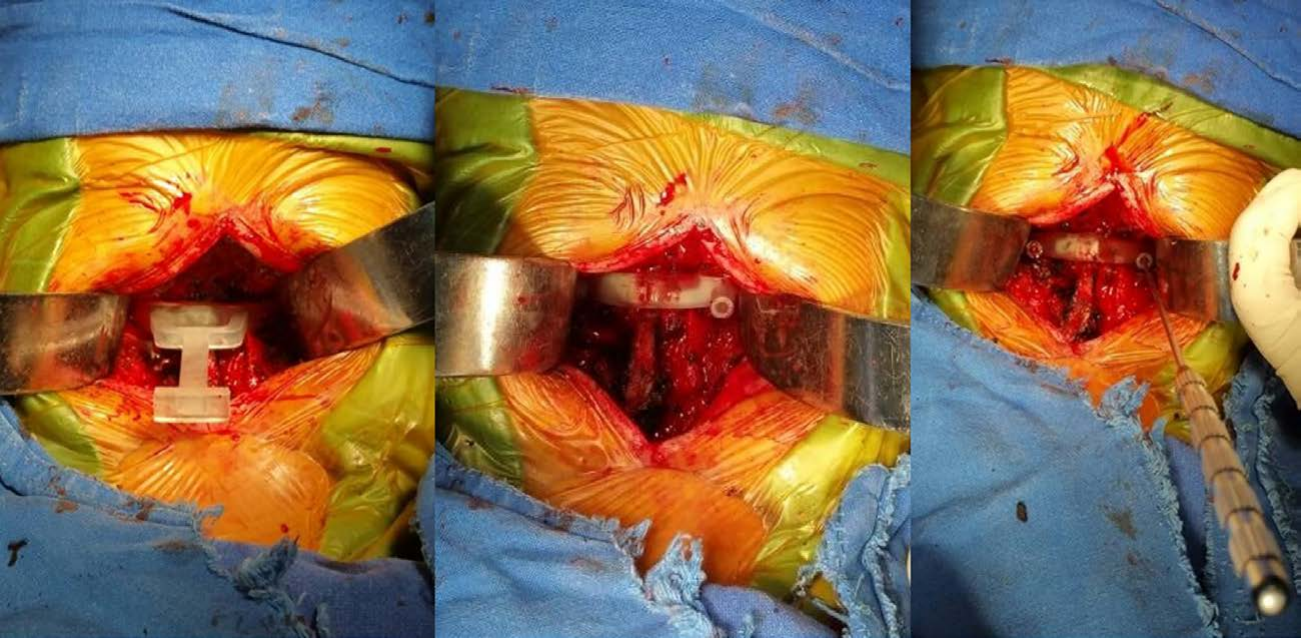
A 3D-printed navigation guide plate was utilized to assist screw implantation during the operation.

The patients received standard perioperative antibiotics (cefuroxime 1.5 g) for 30 minutes prior to surgery. Patients were lying flat after general endotracheal anesthesia was induced. Under the external occipital protuberance, a 6- to 8-cm-long midline skin incision was made (Fig. 3). The lower edge of the C1 posterior arch and lateral mass was exposed after the fascia and ligaments were cut away along the midline to reveal the posterior arch of the vertebra. The C1 to C2 venous plexus was kept as safe as possible during the procedure. The posterior arch was covered with the navigational template. After drilling the pedicle screw tracts, the corridors were marked with a blunt k-wire to allow the template to be removed. Following the placement of pedicle screws into the pilot hole created with the customized drill template, the paths were tapped and pedicle screws were installed (Fig. 4). Intraoperative fluoroscopy was used to confirm the proper placement of all screws. The operative time for this patient was 71 minutes, the intraoperative blood loss was 110 mL, and there were no spinal cord or vertebral artery injuries. The procedure went off without a hitch.

**Figure 5.**
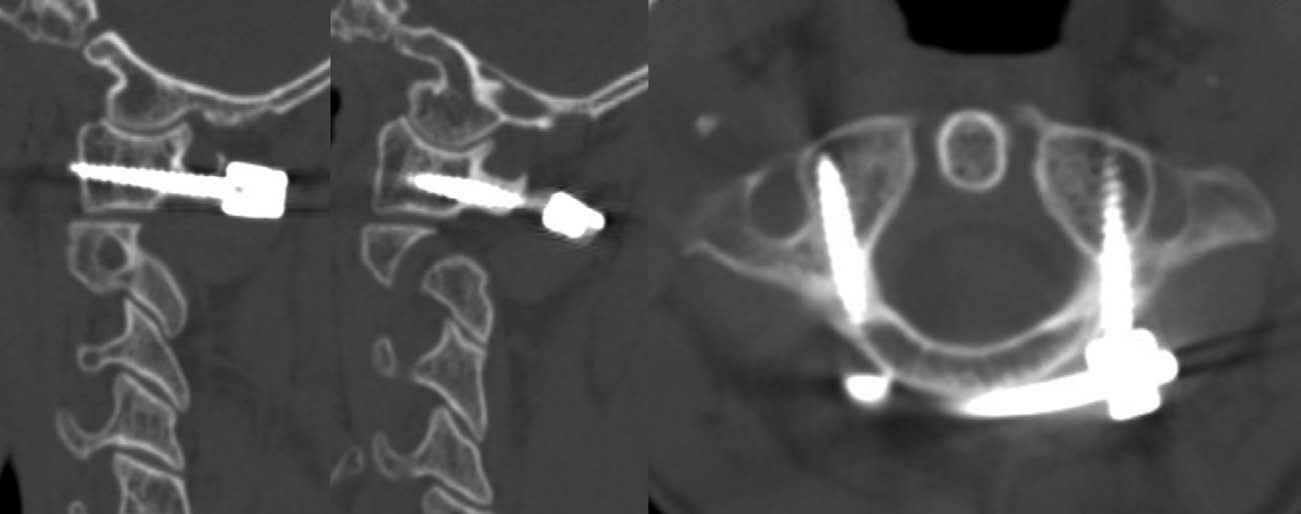
Axial and sagittal view of CT scan taken 3 days postoperatively showing appropriate positioning of the C1 pedicle screws. CT = computed tomography.

**Figure 6.**
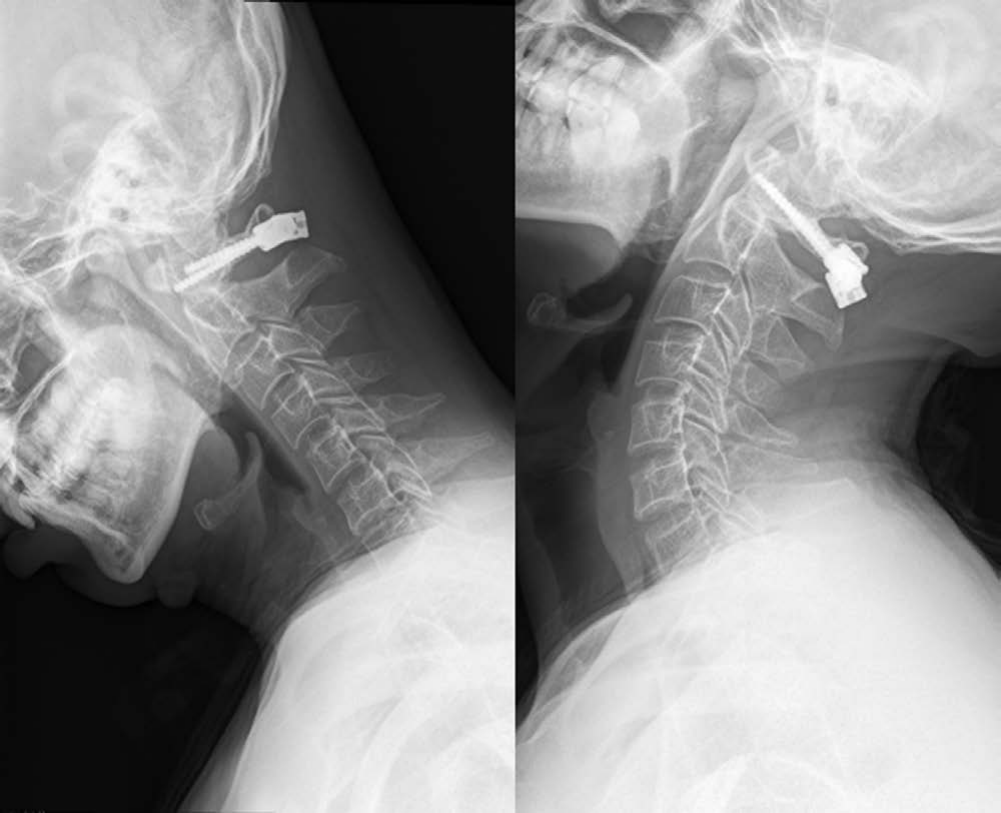
The flexion-extension radiographs at the 12-month follow-up reveal normal cervical alignment, no implant failure, and no instability at C1 to C2.

**Figure 7.**
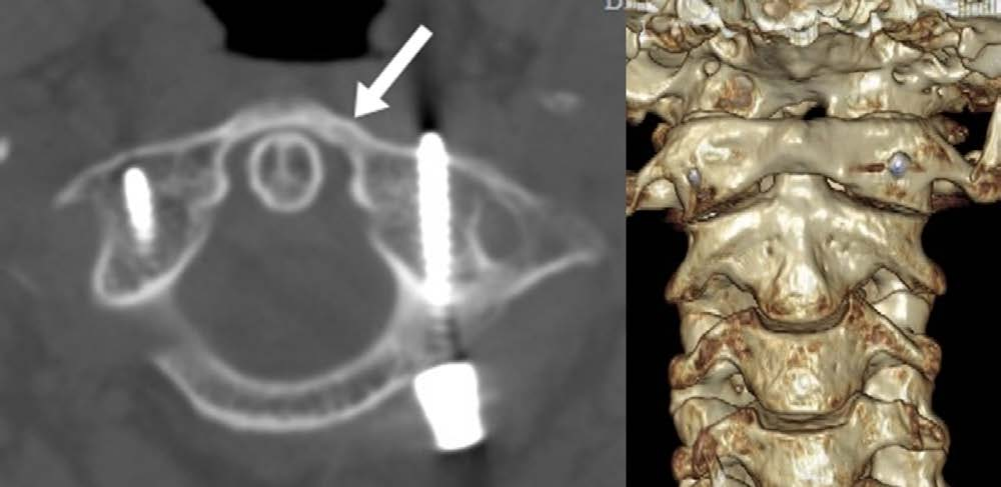
A year following surgery, a CT scan and 3-dimensional reconstruction revealed that the C1 fracture had healed and the cervical spine was in good alignment. CT = computed tomography.

Axial and sagittal views of a CT scan taken 3 days after surgery, demonstrating proper positioning of the C1 pedicle screws (Fig. 5). Antibiotics were continued for 2 days. With a Philadelphia cervical collar, the patient was able to get out of bed on the first day after surgery. The patient recovered smoothly and was discharged 1 week later. He continued to receive external immobilization for 12 weeks followed by a gradual return to full activities. Follow-up X-ray examination at 1 month and 2 months, radiographs and CT scanning at 3 months and 12 months. At 12 months, flexion-extension radiographs revealed normal cervical alignment in this patient, with no implant failure or C1 to C2 instability (Fig. 6). The 1-year postoperative CT scan revealed satisfactory cervical alignment with no implant failure and healing of the C1 fractures (Fig. 7). The patient expressed no discomfort and refused to have the internal fixation surgically removed. The average VAS score decreased from 8 before surgery to 2 at 12 months. Informed consent was obtained from the patient for the publication of this case report details.

## 3. Discussion

This study reported the first case of treating unstable C1 semi-ring fractures (Landells type II) with direct C1 pedicle screw fixation using a navigational template. The management of C1 fractures is controversial. Historically, C1 to C2 or C0 to C2 fusion or external immobilization have been used to treat atlas fractures. According to recent literature-based evaluations of C1 ring fractures treated nonoperatively (including semi-ring fractures of Landells type II), like with the Halovest or rigid brace, failure to establish a nonunion can lead to chronic discomfort, cervical muscle spasms, and limited neck range of motion.^[1,6]^ This just proves the rationality of internal fixation treatment for this patient. C1 to C2 or C0 to C2 fusion has traditionally been used to treat unstable C1 semi-ring fractures due to concern over C1 to C2 instability. Conventional definitions of C1 instability that place a focus on TAL integrity overestimate the number of fractures that call for C1 to C2 fusion and understate the number of fractures that call for operative intervention.^[3]^ A common treatment for unstable atlas fractures is C1 to C2 or C0 to C2 fusion, but this involves significant motion loss, specifically in the C1 to C2 rotation and C0 to C1 flexion extension.^[7]^ Thus, the patient refused to perform atlantoaxial fusion surgery. According to studies, C1 direct posterior internal fixation can provide atlas fractures instant stabilization while preserving the movement of the occipital atlantoaxial joint.^[2]^ Direct posterior C1 pedicle screw fixation and lateral mass screw internal fixation are 2 C1 fixation methods. C1 pedicle screw fixation has been shown to have better biomechanical stability than the other 2 options and can lessen the risk of C1 to C2 venous plexus bleeding.^[8]^

However, we deemed that C1 pedicle screw insertion was technically challenging because of the variations in individual anatomy and the slim margin for error when inserting screws into a narrow bony channel. The conventional approach to inserting C1 pedicle screws relies on meticulous preoperative image analysis and familiarity with anatomic landmarks to prevent incorrect placement.^[9]^ Cortical pedicle perforation still happens despite the fact that numerous techniques that claim to improve pedicle screw placement accuracy have been described. Therefore, we have developed and used a navigational template of a personalized drill template to ensure the accuracy of C1 pedicle screw placement in order to overcome challenges brought on by differences in individual anatomy and reduce the risk of injury to the spinal cord, vertebral artery, and internal carotid artery. The template is made to rest against the bony architecture of the C1 posterior arch, matching it to create a complementary fit. The stability of this fit is essential because even minor fit imperfections could cause the C1 screws to deviate from their intended course. There is no evidence, according to earlier research, that the screw’s position after being installed using the template deviates significantly from the intended trajectory.^[10]^ Finally, all of the fractures were successfully united, and the patient’s clinical symptoms improved without any neurological deficits.

This case shows the precision of navigational template technology and its capacity to produce customized drill templates that are accurate even when dealing with relatively uniform bony surfaces, like the posterior elements of C1. Success at C1 was probably aided by additional surgical methods, such as drilling the second pilot hole while keeping the drill bit on the first side to stabilize the template. In addition, a number of additional factors, such as inadequately thin CT scan cuts, human error when using the tools or software mentioned earlier, and technical complications during surgery, may affect the accuracy of navigational template technology.

To ensure a perfect fit between the template and the bone, care must be taken to remove all soft tissues from the posterior spinal elements during surgery. To ensure that the drill remains in the C1 pedicle, it is crucial that the surgeon closely follows the drill guide’s instructions and moves in 1-mm increments. The use of a navigational template necessitates more soft tissue removal from the C1 posterior arch when compared to conventional technique. This drawback, though, is probably considerably less gruesome than a lost C1 pedicle screw. When the C1 posterior arch is significantly displaced, this navigational template technique cannot be used because the template cannot stay stable on the C1 posterior arch. This case shows how, in the appropriate clinical setting, the use of a navigational template can improve the accuracy of posterior C1 pedicle screw fixation of C1 ring fractures.

## 4. Conclusion

In patients with C1 semi-ring fractures, this case shows that it is technically possible to use a navigational template to help with pedicle screw placement. If this technology is widely adopted in clinical practice, the increased screw placement accuracy and decreased pedicle breeches may result in clinically significant improvements in surgical care. Additionally, this technique may be particularly useful for surgeons who have less experience inserting C1 pedicle screws by hand.

## Author contributions

Data curation: Wei-Xin Dong.

Formal analysis: Zhen-Tao Chu.

Investigation: Wei-Xin Dong, Zhen-Tao Chu.

Writing – original draft: Wei-Xin Dong.

Writing – review & editing: Yong Hu, Wei-Xin Dong, Zhen-Tao Chu.
